# Anti-SARS-CoV-2 Antibodies versus Vaccination Status in CAD Patients with COVID-19: A Prospective, Propensity Score-Matched Cohort Study

**DOI:** 10.3390/vaccines12080855

**Published:** 2024-07-30

**Authors:** Sylvia Mink, Heinz Drexel, Andreas Leiherer, Janne Cadamuro, Wolfgang Hitzl, Matthias Frick, Patrick Reimann, Christoph H. Saely, Peter Fraunberger

**Affiliations:** 1 Central Medical Laboratories, 6800 Feldkirch, Austria; 2 Private University in the Principality of Liechtenstein, 9495 Triesen, Liechtenstein; 3 VIVIT Institute, Academic Teaching Hospital Feldkirch, 6800 Feldkirch, Austria; 4 Drexel University College of Medicine, Philadelphia, PA 19129, USA; 5 Department of Laboratory Medicine, Paracelsus Medical University, 5020 Salzburg, Austria; 6 Department of Research and Innovation, Team Biostatistics and Publication of Clinical Trials, Paracelsus Medical University, 5020 Salzburg, Austria; 7 Department of Internal Medicine, Academic Teaching Hospital Feldkirch, 6800 Feldkirch, Austria

**Keywords:** COVID-19, vaccination, anti-SARS-CoV-2 spike antibodies, SARS-CoV-2, inflammation, correlate of protection

## Abstract

Objectives: Despite the currently prevailing, milder Omicron variant, coronary artery disease (CAD) patients constitute a major risk group in COVID-19, exhibiting 2.6 times the mortality risk of non-CAD patients and representing over 22% of non-survivors. No data are currently available on the efficacy of antibody levels in CAD patients, nor on the relevance of vaccination status versus antibody levels for predicting severe courses and COVID-19 mortality. Nor are there definitive indicators to assess if individual CAD patients are sufficiently protected from adverse outcomes or to determine the necessity of booster vaccinations. Methods: A prospective, propensity-score-matched, multicenter cohort study comprising 249 CAD patients and 903 controls was conducted. Anti-SARS-CoV-2-spike antibodies were measured on hospital admission. Prespecified endpoints were in-hospital mortality, intensive care, and oxygen administration. Results: After adjustment for potential confounders, CAD patients exhibited 4.6 and 6.1-times higher mortality risks if antibody levels were <1200 BAU/mL and <182 BAU/mL, respectively, compared to CAD patients above these thresholds (aOR 4.598, 95%CI 2.426–8.714, *p* < 0.001; 6.147, 95%CI 2.529–14.941, *p* < 0.001). Risk of intensive care was 3.7 and 4.0 (*p* = 0.003; *p* < 0.001), and risk of oxygen administration 2.6 and 2.4 times higher below these thresholds (*p* = 0.004; *p* = 0.010). Vaccination status was a weaker predictor of all three outcomes than both antibody thresholds. Conclusion: Antibody levels are a stronger predictor of outcome in CAD patients with COVID-19 than vaccination status, with 1200 BAU/mL being the more conservative threshold. Measuring anti-SARS-CoV-2 antibodies in CAD patients may ensure enhanced protection by providing timely booster vaccinations and identifying high-risk CAD patients at hospital admission.

## 1. Introduction

As of March 2024, severe acute respiratory syndrome coronavirus 2 (SARS-CoV-2) has affected over 775 million individuals [1], with over 7 million COVID-19-related deaths worldwide. The official end of the COVID-19 public health emergency of international concern was announced in May 2023 [2]. Since then, testing for SARS-CoV-2 has substantially decreased, and infection numbers are no longer reported in detail [3], which suggests there may be a considerable number of unreported cases. The persistence of SARS-CoV-2 in the human population is also visible through wastewater monitoring, which has been useful as an indirect tool to assess community-level circulation [4,5]. In addition, new SARS-CoV-2 variants, such as JN.1, a descendant of BA2.86, and a new variant of interest with an additional mutation in its spike protein, continue to be identified [6,7]. Given the persistence of SARS-CoV-2 in the human population and the continued emergence of new variants, COVID-19 is going to remain a relevant concern with regard to vulnerable patient groups [8,9].

A history of coronary artery disease (CAD) has been linked to severe courses and elevated mortality rates in COVID-19 [10,11], with up to 22.9% of non-survivors having been diagnosed with CAD [12]. Accordingly, higher rates of intensive care unit (ICU) admission, severe COVID-19, and 28-day in-hospital mortality have been reported in CAD patients [11]. In a retrospective study of 457 CAD patients, the degree of coronary artery calcification was a predictor of increased rates of ICU admission, mechanical ventilation, length of hospital stay, in-hospital, and 30-day mortality [13]. Mortality rates ranged from 8.6% in patients without coronary artery calcification to 27.3% in patients with severe calcification [13]. In a recent meta-analysis comprising close to 19,000 patients, the odds of COVID-19 mortality were estimated to be 2.6 times higher for CAD patients (pooled OR 2.64, 95%CI 2.30–3.04) [11].

Concurrently, COVID-19 has been described to both exacerbate subclinical pre-existing cardiovascular problems and cause de novo myocardial damage, which may, in turn, increase mortality risk [10]. The pathophysiological mechanisms have not been elucidated, but concordant increases of troponins and inflammatory markers such as interleukin-6, C-reactive protein (CRP), and D-dimer have suggested systemic inflammation and secondary hemophagocytic lymphohistiocytosis as a potential cause [14]. Other potential mechanisms include stress cardiomyopathy, increased thrombogenicity, and myocarditis [14]. However, while examinations of cardiac tissue samples from COVID-19 deaths showed high rates of myocardial ischemia, thrombosis, and cardiac dilatation, changes consistent with myocarditis were only present in 1.5% of cases [10,15].

SARS-CoV-2 attaches to host cells by means of the angiotensin-converting enzyme (ACE)-2 receptor [16]. As ACE2 is abundantly expressed in alveolar epithelial cells and highly expressed in vasculature, including arterial and venous endothelial cells as well as arterial smooth muscle cells, ACE2 is suspected to contribute to cardiovascular complications on a molecular level [16].

In a recent systematic review comprising over 30 million individuals, antibody levels were indirectly linked to risk of SARS-CoV-2 infection, severe courses, and adverse outcomes, most notably COVID-19 mortality. Further, an incremental increase in protection with increasing antibody levels was noted [17].

However, antibody responses to vaccinations and preceding infections exhibit considerable variability between individuals [18,19]. In comparison to non-CAD COVID-19 patients, CAD patients are, on average, significantly older [10,11], which may negatively affect outcomes due to increased frailty and the progressive accumulation of comorbidities [20,21]. In addition, previous studies indicate that advancing age is associated with poorer antibody production; a faster decline in antibody levels following vaccination; and reduced antibody affinity, attributable to reduced somatic hypermutation, decreased frequency of isotype switching, and lower occurrence of spontaneous mutations within variable regions [22,23,24,25].

At present, there are no definitive indicators available to determine if individual CAD patients are sufficiently protected from severe COVID-19 and COVID-19-related mortality. Accordingly, there is currently no tool for guiding the timing and frequency of booster vaccinations in CAD patients [26].

Insufficient data exist regarding the efficacy of antibody levels in patients diagnosed with coronary artery disease [17]. There is also a lack of data evaluating the significance of vaccination status compared to antibody levels in predicting severe outcomes and COVID-19-related mortality in this high-risk patient population. Further, no data have been presented to establish a specific threshold of protective antibodies in CAD patients, by which the necessity of booster vaccinations may be determined.

This prospective, propensity score-matched, multicenter cohort study, comprising 249 CAD patients and 903 controls, therefore, provides data on the clinical utility of measuring anti-SARS-CoV-2 spike antibodies in this important patient subset. This study further evaluates the relevance of antibody levels compared to vaccination status as a correlate of protection against COVID-19 in hospitalized CAD patients, non-CAD patients, and matched controls.

## 2. Methods

### 2.1. Study Design and Participants

In this prospective, propensity score-matched, multicenter cohort study, we consecutively enrolled hospitalized COVID-19 patients from five hospitals in Austria between 1 August 2021 and 10 April 2022. Patients were followed until 90 days after hospital admission.

Prerequisites for inclusion were a positive polymerase chain reaction (PCR)-based SARS-CoV-2 test result and the collection of a venous blood sample on hospital admission. A preceding hospital admission during the study period, insufficient sample material, or not having been discharged by the end of the study period were causes for exclusion.

Sample size calculation was conducted for cohort studies with dichotomous outcomes and independent proportions [27,28] with correction for continuity [27]. Parameters were defined as follows: type I error rate α (two-sided significance level) of 0.05, statistical power of 0.8, expected dropout rate of 10%, and a ratio between groups of 1. Based on mortality rates at the beginning of the study [29], we estimated mortality rates of 10% and 20% for patients with high and low antibody levels. This yielded a minimum required sample size of 486 patients.

### 2.2. Variables

The main outcome of this investigation was all-cause in-hospital mortality. Secondary endpoints encompassed intensive care unit (ICU) admission and oxygen administration for respiratory support. Patients were categorized as fully vaccinated against SARS-CoV-2 if they had received either one dose of an accepted single-dose vaccine or two doses of an accepted two-dose vaccine. CAD patients were classified by ICD-10 diagnosis as obtained from patient records.

Established risk factors associated with severe COVID-19 courses and increased mortality rates were selected as predefined covariates. These risk factors encompassed age; body mass index (BMI); SARS-CoV-2 variant; and severe comorbidities, including type II diabetes, hypertension, heart failure, chronic obstructive pulmonary disease (COPD), and renal disease [30,31,32,33,34,35]. Age is considered a primary risk factor for disease severity and COVID-19 mortality, owing to a combination of general frailty and the progressive accumulation of comorbidities [20]. Different SARS-CoV-2 variants have also been closely linked to outcomes, with lower mortality rates for the Omicron compared to the Delta variant [29,36].

### 2.3. Data Sources and Measurements

Anti-SARS-CoV-2 spike antibodies, interleukin 6, C-reactive protein (CRP), creatinine, and NT-proBNP were measured on Roche Cobas 6000 or 8000 systems, employing the Elecsys Anti-SARS-CoV-2-S assay, the Elecsys IL-6 assay, the CRP4 assay, the creatinine Jaffé Gen.2 assay, and the Elecsys proBNP II assay, respectively.

Clinical data on patient characteristics, vaccination history, disease course, and outcome were extracted from patient records. In particular, we recorded age, gender, BMI, vaccination status and type of vaccine, reason for hospitalization, symptoms present at hospital admission, number of days in the hospital, main diagnosis, oxygen administration, number of days in intensive care, as well as in-hospital mortality. SARS-CoV-2 variant and PCR-derived cycle threshold values were also noted.

### 2.4. Ethical Approval

The local Institutional Review Board (IRB), Ethikkommission Vorarlberg, Roemerstrasse 15, A-6901 Bregenz, approved the study. This study was carried out in accordance with the Declaration of Helsinki of 1975 (revised 2013) and Good Clinical Research Practice.

### 2.5. Statistical Methods

The IBM Statistical Package for the Social Sciences (SPSS), version 29 (IBM, Armonk, NY, USA), was used for conducting all statistical analyses. Descriptive statistical parameters included frequencies, percentages, means, 95% confidence intervals for means, medians, standard deviations, and interquartile ranges. Mann–Whitney U tests and Kruskal–Wallis tests were utilized to evaluate the statistical significance of continuous variables, while chi-square tests were employed for categorical variables, as appropriate. A two-sided *p*-value of <0.05 was classified as statistically significant.

As CAD patients were found to differ significantly from non-CAD patients in age and comorbidities, propensity score-matching was conducted to improve comparability. Covariates for propensity score-matching were selected based on potential relations to outcome and CAD diagnosis as well as on significant differences between the two groups. Patients were matched by age, SARS-CoV-2 variant, type 2 diabetes, hypertension, and renal disease [20,21,29,33] using a maximum allowable difference in propensity score of 0.1 and replacement. Propensity scores were then evaluated for common support, and overlap was found to be satisfactory. Balance was achieved in all matched covariates but not in the parameters heart failure, CVD, and comorbidity count. Nonetheless, the differences between these groups were found to have been notably reduced as well (see Table 1).

In order to assess the risk associated with lower levels of anti-SARS-CoV-2 spike antibodies, we constructed multiple logistic regression models for each endpoint: using firstly a threshold of 1200 binding antibody units (BAU)/ml [37], which was obtained by dividing the measuring range in half; secondly, a threshold of 182 BAU/mL as defined by the Youden index; and thirdly, the patient’s vaccination status as comparison.

Prerequisites for logistic regression analysis were verified, including lack of duplicate entries (independence of errors), linear relationships between continuous variables and the logit transformation of the dependent variable (Box–Tidwell test), absence of multicollinearity, and lack of strongly influential outliers.

Regression models were built using a forward stepwise approach, where outcome was input as the dichotomous dependent variable, and predefined covariates were input as independent variables. The following potential confounders were considered: age, BMI, SARS-CoV-2 variant, type II diabetes, hypertension, and renal disease [20,21,29,33]. Only the variables age, BMI, and SARS-CoV-2 variant were found to improve the prediction of the logistic regression models. With regard to the endpoint oxygen administration, the covariate type 2 diabetes was also significant. Odds ratios were reported with 95% confidence intervals (CI).

To provide a further measure of risk, we employed Cox proportional hazard models to estimate hazard ratios for our primary and secondary endpoints. Proportional hazard models were built analogously to the multiple logistic regression models. The additional variable time to event was input as days measured from hospital admission. All risk measures are given with 95% confidence intervals. Cumulative survival over time is graphically depicted using Kaplan–Meier curves and tested for statistically significant differences with log-rank (Mantel-Cox) tests.

In order to verify the robustness of these models, all regression models were rebuilt using a direct model-building approach. Statistically significant independent variables that improved the prediction of multiple logistic and Cox regression models were entered simultaneously with equal weighting (method “enter”) while conducting bootstrapping with 2000 Monte Carlo samples. Finally, goodness of fit was confirmed using Hosmer–Lemeshow tests.

## 3. Results

### 3.1. Participants

**Figure 1:**
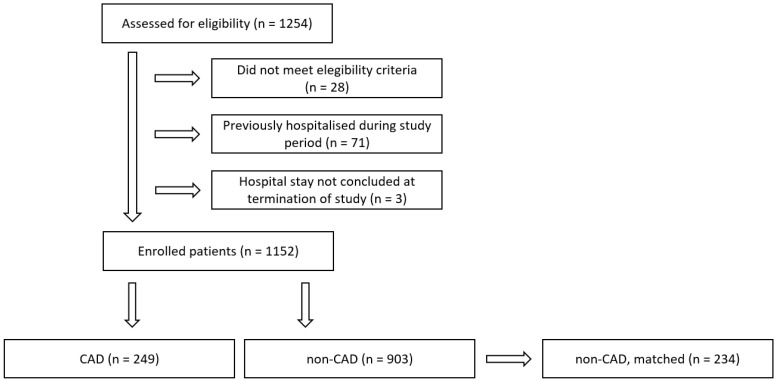
Patient flow diagram. CAD: coronary artery disease.

Between 1 August 2021 and 10 April 2022, 1254 COVID-19 patients who were admitted to one of five hospitals were assessed for eligibility. Following the application of exclusion criteria, the final cohort comprised 1152 patients, including 249 CAD patients and 903 controls. Propensity score-matching yielded 234 matched non-CAD patients. Patient flow is illustrated in Figure 1.

Among CAD patients, 39 patients died, 41 were admitted to an intensive care unit, and 152 required oxygen administration. With regard to controls, we registered 79 deaths, 124 intensive care admissions, and 433 cases of oxygen administration.

Table 1 provides a detailed overview of patient characteristics for the whole study cohort; Table 2 compares patient characteristics of CAD patients by vaccination status, statin treatment, and Omicron variant; and Table 3 shows patient characteristics of CAD vs. non-CAD and matched, non-CAD patients regarding vaccination status and statin treatment.

Of note, CAD patients were, on average, 15 years older than non-CAD patients and exhibited a significantly higher prevalence of comorbidities. Accordingly, in-hospital mortality rates were 1.8 times higher in CAD compared to non-CAD patients.

Propensity score-matching between CAD and non-CAD patients was able to compensate for the difference in age, which is known to be a major risk factor for adverse outcomes and the differences in the prevalence of the majority of comorbidities but could not completely compensate for the varying prevalence of heart failure and cerebrovascular disease.

Following propensity score-matching, the difference in in-hospital mortality rates between CAD and matched non-CAD patients was no longer statistically significant.

### 3.2. Patient Outcome by Antibody Level and Vaccination Status

In order to evaluate the association between COVID-19 in-hospital mortality, intensive care admission, and oxygen requirements with anti-SARS-CoV-2 spike antibody levels, we classified CAD, non-CAD, and matched non-CAD patients into categories of high and low antibody levels using a threshold of 1200 BAU/mL, thus separating the measuring range by half [37].

Aiming to provide an alternative threshold for assessing the association of anti-SARS-CoV-2 spike antibody levels with outcomes in CAD patients, we further employed high and low antibody levels as defined by the Youden index of 182 BAU/mL.

**Figure 2:**
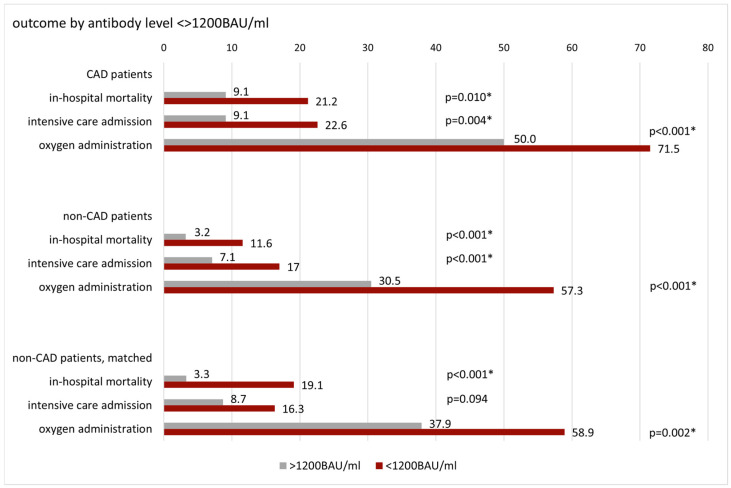

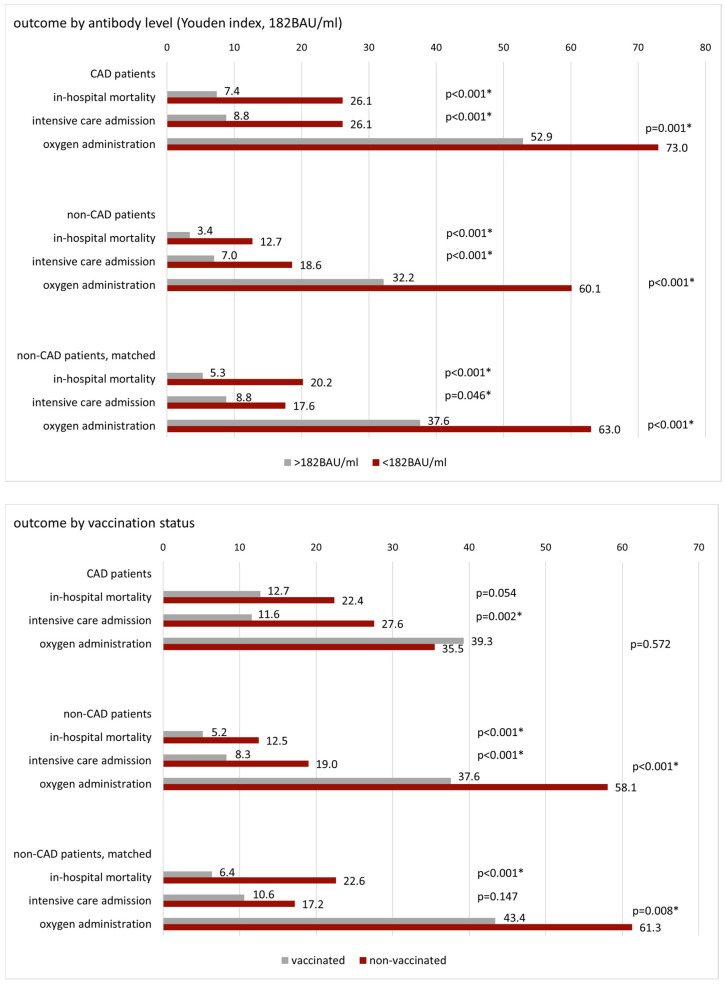
Patient outcomes in percentages regarding in-hospital mortality, intensive care treatment, and oxygen administration by anti-SARS-CoV-2 spike antibody levels <> 1200 BAU/mL; by anti-SARS-CoV-2 spike antibody levels <> 182 BAU/mL (Youden index); and by vaccination status in CAD (coronary artery disease), non-CAD, and matched non-CAD patients. BAU: binding antibody units, CAD: coronary artery disease, * statistically significant.

The results of these measurements were then compared to outcomes by vaccination status to assess the relevance of antibody levels vs. being vaccinated in CAD patients, non-CAD patients, and matched non-CAD patients. Patient outcomes in percentages by antibody level and vaccination status in CAD, non-CAD, and matched non-CAD patients are depicted in Figure 2.

Anti-SARS-CoV-2 spike antibodies were significantly lower in CAD, non-CAD, and matched non-CAD patients who died than in those who survived (CAD: mean 634 BAU/mL, 95%CI: 285–983 BAU/mL vs. 1254 BAU/mL, 1093–1414 BAU/mL, *p* < 0.001; non-CAD: 359 BAU/mL, 172–546 BAU/mL vs. 939 BAU/mL, 860–1018 BAU/mL, *p* < 0.001; non-CAD matched: 344 BAU/mL, 56–631 BAU/mL vs. 1133 BAU/mL, 969–1297 BAU/mL, *p* < 0.001).

CAD patients with antibody levels <1200 BAU/mL had a 2.7 times higher risk of in-hospital mortality than CAD patients above this threshold (OR: 2.685, 95%CI: 1.245–5.791, *p* = 0.010). The difference in mortality risk was even higher for CAD patients below vs. above 182 BAU/mL (OR: 4.456, 95%CI: 2.062–9.631, *p* < 0.001). In contrast, the mortality risk of CAD patients did not differ significantly by vaccination status.

CAD patients exhibited higher mortality rates than non-CAD patients, both in patients with low antibody levels and in patients with high antibody levels, regardless of antibody threshold. After propensity score-matching, mortality rates of CAD patients no longer differed significantly from matched non-CAD patients, either overall or across categories of low and high antibody titers.

With regard to secondary outcomes, anti-SARS-CoV-2 spike antibodies were also found to be significantly lower in CAD patients who required intensive care admission or oxygen administration than in those who did not (ICU mean: 648 BAU/mL, 95%CI: 310–987 BAU/mL vs. 1257 BAU/mL, 1095–1418 BAU/mL, *p* < 0.001; oxygen administration: 944 BAU/mL, 763–1126 BAU/mL vs. 1500 BAU/mL, 1259–1741 BAU/mL, *p* = 0.001).

In CAD patients, risk of ICU admission increased by 2.9 and 3.6 times (OR 2.925, 95%CI: 1.363–6.274, *p* = 0.004; OR: 3.654, 95%CI: 1.764–7.570, *p* < 0.001) and risk of oxygen administration by 2.5 and 2.4 times (OR: 2.513, 95%CI: 1.484–4.255, *p* < 0.001; OR: 2.400, 95%CI: 1.402–4.108, *p* = 0.001) if antibody levels were below 1200 BAU/mL and 182 BAU/mL, respectively. As was observed for our primary outcome in-hospital mortality, the risk of oxygen administration did not differ significantly by vaccination status. However, risk of ICU admission was 2.9-fold higher in non-vaccinated compared to vaccinated CAD patients (OR: 2.921, 95%CI: 1.472–5.797, *p* = 0.002).

### 3.3. Survival over Time

**Figure 3:**
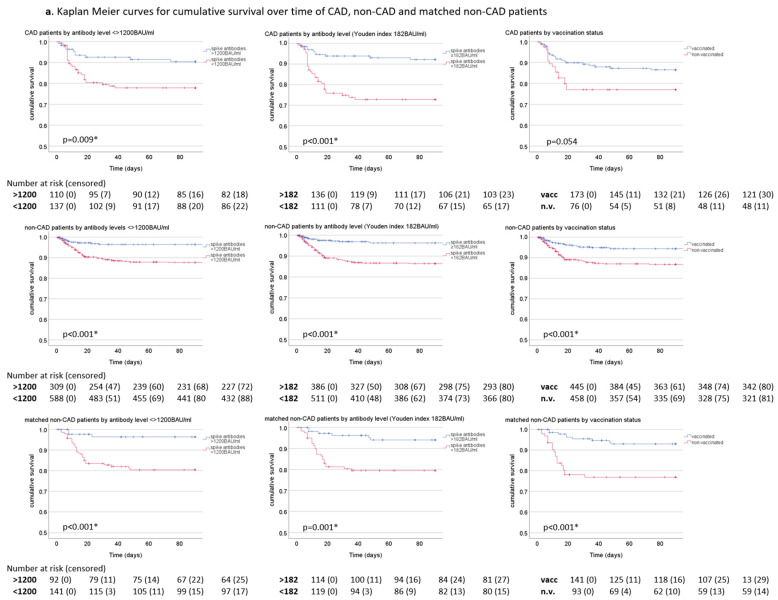

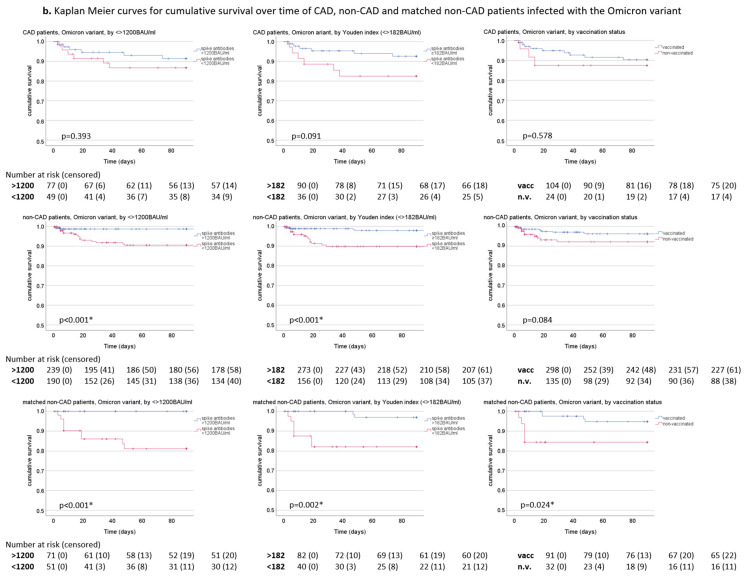
(**a**). Kaplan–Meier curves for cumulative survival over time in CAD (top row), non-CAD (middle row), and matched non-CAD patients (bottom row) by anti-SARS-CoV-2 spike antibody level above vs. below 1200 BAU/mL, by anti-SARS-CoV-2 spike antibody levels above vs. below the Youden index of 182 BAU/mL, and by vaccination status. (**b**) Kaplan–Meier curves for cumulative survival over time in CAD, non-CAD, and matched non-CAD patients infected with the Omicron variant. Statistical significance was determined by log-rank (Mantel-Cox) test. Numbers at risk and cumulative number of patients lost to follow-up (censored) are shown below the graphs. BAU: binding antibody units, CAD: coronary artery disease, spike antibodies: anti-SARS-CoV-2 spike antibodies, vacc.: vaccinated patients, n.v.: non-vaccinated patients, *: statistically significant.

Figure 3 shows Kaplan–Meier curves of cumulative survival over time for CAD patients, non-CAD patients, and matched CAD patients, stratified by antibody level <> 1200 BAU/mL, <> 182 BAU/mL (Youden index) and by vaccination status.

Cumulative survival over time was significantly higher in CAD patients with higher anti-SARS-CoV-2 spike antibody levels compared to CAD patients with lower antibody levels (<>1200 BAU/mL *p* = 0.009, <> 182 BAU/mL *p* < 0.001). In contrast, cumulative survival of CAD patients did not differ significantly by vaccination status. Further, both CAD patients with antibody levels >182 BAU/mL or >1200 BAU/mL exhibited higher cumulative survival than vaccinated CAD patients.

In CAD patients with antibody levels <182 BAU/mL, cumulative survival was slightly lower than in CAD patients with antibody levels <1200 BAU/mL. In contrast, cumulative survival was comparable in CAD patients >182 BAU/mL and >1200 BAU/mL.

Non-CAD patients both above and below 1200 BAU/mL showed significantly higher cumulative survival than CAD patients (*p* = 0.003, *p* = 0.021). However, after propensity score-matching, the difference in survival was no longer statistically significant.

Of note, vaccinated CAD, non-CAD, and matched non-CAD patients showed slightly lower cumulative survival than patients with antibody levels >1200 BAU/mL in the same categories.

Similar results were observed for patients infected with the currently prevailing Omicron variant (Figure 3b). As in the whole cohort, cumulative survival was higher in non-CAD patients than in CAD patients but comparable after propensity score-matching. CAD patients infected with the Omicron variant showed better cumulative survival if antibody levels were >1200 BAU/mL or >182 BAU/mL, albeit not at a statistically significant level. Results were, however, significant for both non-CAD and matched non-CAD patients infected with the Omicron variant. Both groups had significantly better cumulative survival with higher antibody levels, regardless of antibody threshold. Survival over time did not differ significantly by vaccination status in CAD and non-CAD patients infected with the Omicron variant.

### 3.4. Risk Estimation and Adjustment for Potential Confounders

To evaluate the risk linked with lower levels of anti-SARS-CoV-2 spike antibodies, we constructed multiple logistic regression models for all outcomes. Additionally, we computed Cox proportional hazard models for the primary endpoint in-hospital mortality to offer an additional measure of risk that also accounts for the time to death. In order to mitigate the impact of potential confounding factors, these models were subsequently adjusted for the covariates age, BMI, SARS-CoV-2 variant, hypertension, heart failure, and renal disease.

**Figure 4:**
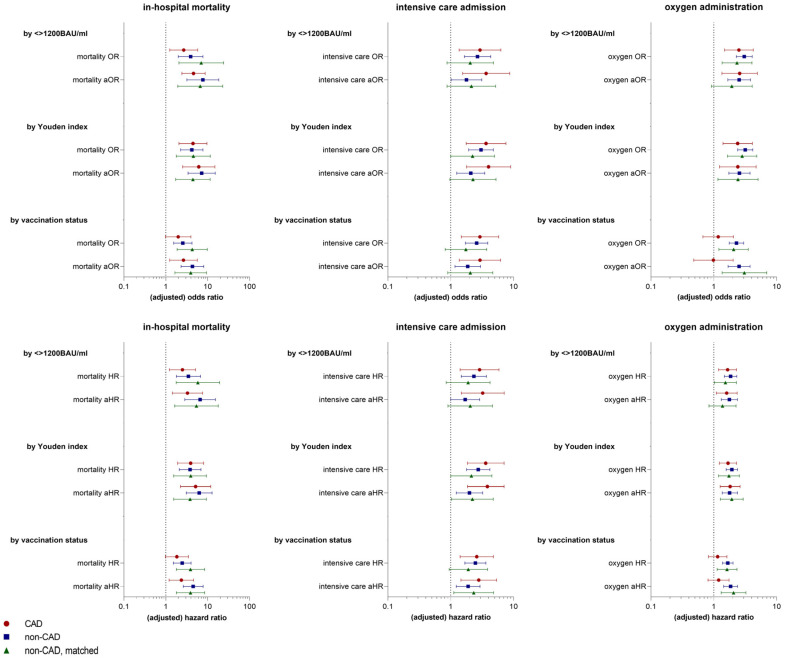
Risk of in-hospital mortality, intensive care admission, and oxygen administration with 95%CI in patients with CAD (coronary artery disease), patients without CAD (non-CAD), and matched patients without CAD (non-CAD, matched). Outcomes are shown by anti-SARS-CoV-2 spike antibody level <> 1200 BAU/mL, by anti-SARS-CoV-2 spike antibody level <> 182 BAU/mL (Youden index), and by vaccination status. Top row: unadjusted and adjusted odds ratios. Bottom row: unadjusted and adjusted hazard ratios. Adjusted odds and hazard ratios were adjusted for age, BMI, SARS-CoV-2 variant, hypertension, heart failure, and renal disease. (a)OR: (adjusted) odds ratio, (a)HR: (adjusted) hazard ratio, BAU: binding antibody unit, CAD: coronary artery disease.

Figure 4 compares both adjusted and unadjusted risk estimates for the outcomes in-hospital mortality, intensive care admission, and oxygen administration in CAD patients, non-CAD, and matched, non-CAD patients.

Following adjustment for potential confounders, CAD patients exhibited 4.6 times and 6.1 times higher mortality risks if antibody levels were <1200 BAU/mL and <182 BAU/mL, respectively, compared to CAD patients above these thresholds (aOR: 4.598, 95%CI: 2.426–8.714, *p* < 0.001; aOR: 6.147, 95%CI: 2.529–14.941, *p* < 0.001). Further, risk of intensive care admission was approximately 3.7 and 4.0-fold higher (aOR: 3.666, 95%CI: 1.547–8.686, *p* = 0.003; aOR: 4.005, 95%CI: 1.784–8.987, *p* < 0.001), and risk of oxygen requirement 2.6 and 2.4 times higher below these thresholds (aOR: 2.580, 95%CI: 1.346–4.945, *p* = 0.004; aOR: 2.414, 95%CI: 1.233–4.726, *p* = 0.010).

Vaccination status was a weaker predictor of all three outcomes than both antibody thresholds and did not reach statistical significance for the endpoint oxygen requirement (mortality aOR: 2.646, 95%CI: 1.239–5.650, *p* = 0.012; ICU aOR: 2.924, 95%CI: 1.368–6.252, *p* = 0.006).

In the Cox proportional hazards analysis, adjusted risk of death in CAD patients was 3.3 times higher if antibody levels were <1200 BAU/mL and 5.2 times higher if antibody levels were <182 BAU/mL than in CAD patients with antibody levels above these thresholds (aHR: 3.298, 95%CI: 1.436–7.576, *p* = 0.005; aHR: 5.152, 95%CI: 2.242–11.838, *p* < 0.001).

Analogously to multiple logistic regression analysis, vaccination status remained a weaker predictor of all three outcomes in the Cox proportional hazard analysis, with oxygen administration again being below the statistical significance level (mortality aHR: 2.352, 95%CI: 1.199–4.614, *p* = 0.013; ICU aHR: 2.799, 95%CI: 1.456–5.381, *p* = 0.002).

### 3.5. Mortality Risk Estimation by Antibody Titer Increment

To further evaluate a potential dose–effect relation between anti-SARS-CoV-2 spike antibodies and in-hospital mortality risk in CAD patients, we computed the rise in mortality risk linked to decreasing antibody levels in intervals of 250 BAU/mL or one tenth of the measuring range.

After controlling for potential confounding factors, including age, BMI, SARS-CoV-2 variant, hypertension, and renal disease, mortality risk rose by a factor of 1.2 for every 250 BAU/mL decrement in antibody levels (aOR: 1.176, 95%CI: 1.067–1.297, *p* = 0.001). Results were comparable in both non-CAD patients and matched, non-CAD patients (aOR: 1.206, 95%CI: 1.110–1.311, *p* < 0.001; aOR: 1.204, 95%CI: 1.065–1.360, *p* = 0.003).

## 4. Discussion

### 4.1. Key Results

In this prospective, propensity score-matched, multicenter cohort study comprising 249 CAD patients hospitalized with COVID-19 and 903 controls, we were able to demonstrate for the first time that anti-SARS-CoV-2 spike antibodies are a strong predictor of outcome in this high-risk patient group. Additionally, antibody levels were a stronger predictor of outcome, including in-hospital mortality, intensive care admission, and oxygen administration, than vaccination status.

CAD patients constitute one of the main risk groups for severe courses and COVID-19-related mortality, exhibiting up to 2.6 times higher mortality risk than non-CAD patients and representing up to 22.9% of non-survivors [10,11,12]. In order to best protect this vulnerable patient group, a correlate of protection needs to be established. Such a measure would aid in identifying individuals at high risk of adverse outcomes, in assessing the current level of protection in a population, and in determining when and at which frequency booster vaccinations are required.

While booster vaccinations have been shown to decrease the risk of breakthrough infections and lower the rates of severe COVID-19 and COVID-19-related mortality [17,38], a high degree of interindividual variation combined with the loss in strength, quality, and durability of antibody responses with progressing age [22,23,24,25] may render generalized recommendations inadequate in the protection of vulnerable patients.

Previous studies suggest that anti-SARS-CoV-2 antibodies are indicative of protection against severe courses and COVID-19-related mortality in the general population as well as in older adults and diabetic patients [17,38,39,40]. However, no data are currently available on the efficacy of antibody levels in CAD patients, nor are there data available evaluating the relevance of vaccination status in comparison to antibody levels for predicting severe courses and COVID-19-related mortality in this important, high-risk patient group [17]. Further, no data have been put forward to help define a protective antibody threshold in CAD patients by which the necessity of booster vaccinations can be determined.

This study, therefore, provides data on the clinical utility of measuring anti-SARS-CoV-2 spike antibodies and assesses the significance of antibody levels versus vaccination status as a correlate of protection in hospitalized CAD patients, non-CAD patients, and matched controls with COVID-19.

### 4.2. Interpretation

In our data, mortality rates of CAD patients were consistently higher in patients with lower antibody levels. Of note, both antibody thresholds (182 BAU/mL and 1200 BAU/mL) were stronger predictors of primary and secondary outcomes than vaccination status.

CAD patients were significantly older, had a significantly higher prevalence of comorbidities, and had significantly higher mortality rates than non-CAD patients. Propensity score-matching improved comparability, with balanced groups across important, independent risk factors, including age, BMI, SARS-CoV-2 variant, and the prevalence of type 2 diabetes, hypertension, and renal disease. While there was still a trend toward higher mortality in CAD patients after propensity score-matching, this difference was no longer statistically significant. While CAD patients showed a significantly higher number of comorbidities than non-CAD patients and matched non-CAD patients, comorbidity count was not found to be a statistically significant variable for outcome in multiple logistic regression analysis.

The difference in mortality risk above vs. below 1200 BAU/mL was slightly more pronounced in non-CAD patients than in CAD patients (OR: 3.918, 95%CI: 1.987–7.724, *p* < 0.001 vs. OR: 2.685, 95%CI: 1.245–5.791, *p* = 0.010). This is consistent with an overall higher mortality risk in CAD patients [10] and suggests a potentially lower beneficial effect of anti-SARS-CoV-2 antibodies in CAD patients compared to non-CAD patients. However, CAD patients with lower antibody levels still exhibited 2.7-fold higher odds of in-hospital mortality than CAD patients with higher antibody levels. It is also worth noting that the difference in mortality risk above vs. below 1200 BAU/mL was less pronounced when comparing CAD to matched non-CAD patients.

With regard to different antibody cutoffs in CAD patients, we observed slightly higher odds ratios for in-hospital mortality using the Youden index (182 BAU/mL) compared to 1200 BAU/mL. However, with regard to secondary outcomes, anti-SARS-CoV-2 antibody levels above vs. below 1200 BAU/mL were a stronger predictor than the Youden index. Previous studies suggest that the protective effect of antibodies increases with increasing antibody levels [17]. This is in accordance with our data showing a 1.2 increase in mortality risk for each 250 BAU/mL decrement in antibody levels. Hence, in clinical practice, the use of 1200 BAU/mL for determining the necessity of booster vaccinations would be more conservative.

In accordance with previous studies [41], CAD patients exhibited significantly higher concentrations of interleukin-6 and CRP than non-CAD patients. This difference was also present after propensity score-matching, indicating higher levels of inflammation in CAD patients. Statin treatment has previously been linked to lower levels of inflammation [42], which was also confirmed by our data, as CAD patients receiving statin treatment showed lower levels of inflammatory markers than CAD patients without statins. With regard to outcome, we observed lower rates of oxygen administration in CAD patients receiving statins compared to those who did not. While there was a trend to lower mortality and intensive care admission in CAD patients taking statins, these differences did not reach statistical significance (Table 2). In addition, statin treatment was not a significant covariate for outcome in multiple logistic regression analysis.

Despite the primary indication for statin use in this patient group, the low percentage of CAD patients taking statins on hospital admission aligns well with previous studies highlighting major gaps in both the achievement of risk-based LDL-C goals and the adherence to statin treatment [43,44,45]. For instance, an observational study of 1334 STEMI patients who underwent percutaneous coronary intervention reported only 48.1% adherence to statins after 12 months [43].

Of note, despite varying levels of inflammatory parameters in CAD compared to non-CAD and matched non-CAD patients, we did not observe a significant difference in viral load between these groups.

### 4.3. Strengths and Limitations

This study has a number of strengths. Firstly, its high recruitment rate substantially reduces the risk of selection bias. Secondly, the study’s primary endpoint, in-hospital mortality, is not subject to clinical judgment and thus not affected by assessment bias, which may occur in softer clinical endpoints [46]. Thirdly, comparability between CAD and non-CAD patients was improved through propensity score-matching. Fourthly, the laboratory parameters used in this study have short turn-around times and are widely available in routine laboratories. In addition, previous studies have shown that the anti-SARS-CoV-2 spike antibody assay used in this study has a high sensitivity for detecting neutralizing antibodies and maintains that sensitivity over time [47]. Fifthly, we provided two distinct measures of risk, as calculated by multiple logistic regression and Cox proportional hazard models. All models were subsequently adjusted for various established risk factors of severe COVID-19 and COVID-19-related mortality, including age; body mass index; SARS-CoV-2 variant; and severe comorbidities, such as type 2 diabetes, hypertension, and renal disease, which may have acted as potential confounders [20,21,29,33].

With regard to limitations, this study enrolled hospitalized patients only, which may restrict its applicability to an outpatient setting. However, given that severe cases of COVID-19 are normally hospitalized, we deliberately chose to focus on this patient group. This study cohort consists of hospitalized patients treated in Austria, potentially limiting its generalizability to populations with varied access to healthcare, vaccination programs, or demographic profiles.

Following propensity score-matching, the patient count in matched non-CAD patients was lower than in the original control group of non-CAD patients, which resulted in wider confidence intervals in multiple logistic and Cox regression analyses. While the results regarding in-hospital mortality were still statistically significant for matched non-CAD patients, the reduced patient number needs to be taken into account. Furthermore, our findings may not be directly applicable to patients undergoing B-cell-depleting therapies, as their ability to produce antibodies is significantly impaired.

Both humoral and cellular immune responses play critical roles in viral clearance. T-cell-mediated immunity, in particular, is critical for identifying SARS-CoV-2 variants, eliminating the virus, and producing long-lasting memory responses [48]. Further, T-cell responses have been postulated to provide some protection in individuals with inadequate antibody responses [48], and the quantity of CD8^+^ T cells has been linked to disease severity. However, compared to antibody levels, measures of cellular immunity are less widely available in routine laboratories and less established as correlates of clinical immunity [49].

Antibody levels measured on hospital admission are a result of both pre-existing antibodies and those generated in response to the current infection. Consequently, prolonged infections prior to hospital admission may lower the prognostic value of antibody levels at the time of admission. In our cohort, median time from symptom onset to hospitalization was 3 days, with an IQR of 1–7 days. In spite of this limitation, it may be anticipated that patients with antibody levels <1200 BAU/mL at hospital admission had comparable or slightly lower antibody levels at the time of infection. Thus, targeting antibody levels above 1200 BAU/mL in CAD patients may be viewed as a conservative threshold.

Emerging SARS-CoV-2 variants have been linked to varying degrees of immune escape from antibodies formed against other preceding variants [6]. As some loss of efficiency against diverging variants may be anticipated [50,51], updated vaccines are required for ensuring optimal protection of vulnerable patients.

COVID-19 has been suggested to contribute to the development of CAD, to exacerbate subclinical cardiovascular issues, and to cause de novo cardiac injury [10,14]. Future studies are warranted to determine whether antibody levels prevent de novo cardiovascular damage in both CAD patients and the general population.

In our study cohort, mortality risk in CAD patients differed significantly by antibody level, both by 182 BAU/mL and the more conservative threshold of 1200 BAU/mL. However, these thresholds require verification in separate cohorts. Importantly, in our study, both antibody thresholds were stronger predictors of outcome in CAD patients than vaccination status.

## 5. Conclusions

Our data suggest that antibody levels are a stronger predictor of outcome in CAD patients with COVID-19 than vaccination status, with 1200 BAU/mL being the more conservative threshold.

These data further support measuring anti-SARS-CoV-2 antibodies in this important patient subset to ensure optimal protection against adverse courses by providing timely booster vaccinations for individuals with inadequate antibody responses.

In addition, measuring antibody levels on hospital admission of CAD patients may aid in identifying patients at high risk of severe courses and COVID-19 mortality.

## Figures and Tables

**Table 1:** Patient characteristics and outcomes for CAD, non-CAD, and matched, non-CAD patients for the whole cohort (left) and for patients infected with the currently prevailing Omicron variant (right). Quantitative results are given as means ± standard deviation or median and interquartile range, as appropriate. BMI: body mass index, DM: diabetes mellitus, CAD: coronary artery disease, COPD: chronic obstructive pulmonary disease, TIA: transient ischemic attack, CVD: cerebrovascular disease, ICU: intensive care unit, CT: cycle threshold, BAU: binding antibody units, bold print: statistically significant.

Study Cohort	Whole Cohort	CAD	Non-CAD	*p*-Value	Non-CAD, Matched	*p*-Value	CAD, Omicron	Non-CAD, Omicron	*p*-Value	Non-CAD, Omicron, Matched	*p*-Value
*N*	1152	249	903		234		128	433		123	
age (years)	66.8 ± 20.3	78.9 ± 11.1	63.5 ± 21.0	**<0.001**	77.2 ± 14.1	0.647	79.8 ± 11.0	63.0 ± 23.2	**<0.001**	78.1 ± 13.4	0.592
male gender (%)	53.2	57.4	52.0	0.132	54.7	0.546	57.0	57.7	0.887	62.6	0.368
BMI (kg/m^2^)	27.1 ± 6.5	27.0 ± 5.8	27.1 ± 6.7	0.688	26.8 ± 5.0	0.596	26.9 ± 6.2	25.8 ± 6.0	0.091	25.7 ± 5.1	0.107
obesity (%)	25.2	25.7	25.0	0.849	20.9	0.251	23.7	19.7	0.362	14.4	0.091
statin treatment (%)	25.7	48.2	19.5	**<0.001**	28.2	**<0.001**	59.4	24.0	**<0.001**	32.5	**<0.001**
Comorbidities											
DM (%)	24.8	42.4	20.0	**<0.001**	46.6	0.359	40.5	20.6	**<0.001**	51.2	0.093
hypertension (%)	50.5	77.9	43.0	**<0.001**	76.5	0.711	79.7	44.8	**<0.001**	78.9	0.872
heart failure (%)	7.2	20.5	3.5	**<0.001**	5.6	**<0.001**	19.5	3.0	**<0.001**	4.9	**<0.001**
COPD (%)	9.6	14.5	8.3	**0.004**	9.4	0.088	14.8	8.5	**0.037**	8.1	0.096
asthma (%)	2.4	2.0	2.5	0.625	1.3	0.532	2.3	1.2	0.319	0.8	0.333
renal disease (%)	23.1	46.3	16.7	**<0.001**	37.6	0.053	53.2	18.4	**<0.001**	39.0	**0.025**
stroke/TIA/CVD (%)	11.7	24.5	8.2	**<0.001**	14.5	**0.006**	29.7	11.3	**<0.001**	18.7	**0.042**
comorbidity count	1.5 ± 1.5	3.3 ± 1.2	1.0 ± 1.1	**<0.001**	1.9 ± 1.2	**<0.001**	3.4 ± 1.3	1.1 ± 1.2	**<0.001**	2.0 ± 1.1	**<0.001**
Outcome											
mortality (%)	10.2	15.7	8.7	**0.001**	12.8	0.372	9.4	4.4	**0.030**	7.3	0.556
ICU (%)	14.3	16.5	13.7	0.276	13.2	0.321	13.3	6.4	**0.013**	6.5	0.073
oxygen req (%)	51.0	61.8	48.0	**<0.001**	50.7	**0.014**	48.4	27.5	**<0.001**	31.4	**0.006**
Laboratory analysis											
CT value	21.3 ± 6.6	20.8 ± 6.5	21.4 ± 6.6	0.097	20.4 ± 6.6	0.578	20.8 ± 6.5	21.0 ± 6.6	0.818	19.7 ± 6.5	0.149
creatinine (mg/dL)	1.0 (0.8–1.3)	1.2 (1.0–1.7)	1.0 (0.8–1.2)	**<0.001**	1.1 (0.9–1.5)	0.014	1.2 (1.0–1.7)	1.0 (0.8–1.3)	**<0.001**	1.2 (1.0–1.6)	0.770
NT-proBNP (pg/mL)	327 (100–1243)	1387 (477–4333)	227 (69–767)	**<0.001**	661 (176–1649)	**<0.001**	1588 (646–4504)	242 (70–831)	**<0.001**	696 (225–6902)	**<0.001**
IL6 (pg/mL)	28.7 (11–67)	39.0 (17–81)	27.2 (1–64)	**<0.001**	24.9 (11–58)	**0.002**	27 (13–213)	18.4 (8–110)	**0.001**	20.2 (9–45)	0.071
CRP (mg/dL)	6.0 ± 6.4	6.6 ± 6.5	5.8 ± 6.4	**0.026**	5.6 ± 6.4	**0.036**	6.5 ± 6.8	4.4 ± 5.9	**<0.001**	4.3 ± 6.3	**<0.001**

**Table 2:** Left: patient characteristics and outcomes for CAD patients by SARS-CoV-2 variant. Middle: patient characteristics and outcomes for CAD patients by vaccination status. Right: patient characteristics and outcomes for CAD patients by statin treatment. Quantitative results are given as means ± standard deviation or median and interquartile range, as appropriate. BMI: body mass index, DM: diabetes mellitus, CAD: coronary artery disease, COPD: chronic obstructive pulmonary disease, TIA: transient ischemic attack, CVD: cerebrovascular disease, ICU: intensive care unit, CT: cycle threshold, BAU: binding antibody units, bold print: statistically significant.

CAD Patients	Omicron	Non-Omicron	*p*-Value	Non-Vaccinated	Vaccinated	*p*-Value	Statin Treatment	No Statin Treatment	*p*-Value
*N*	128	121		76	173		120	129	
age (years)	79.8 ± 11.0	77.8 ± 11.1	0.140	77.9 ± 13.1	79.3 ± 10.2	0.613	77.7 ± 10.2	79.9 ± 11.9	**0.037**
male gender (%)	57.0	57.9	0.896	52.6	59.5	0.310	65.8	49.6	**0.010**
BMI (kg/m^2^)	26.9 ± 6.2	27.1 ± 5.3	0.594	26.3 ± 5.4	27.3 ± 5.9	0.505	27.7 ± 5.4	26.3 ± 6.1	0.088
obesity (%)	23.7	27.6	0.498	20.6	27.8	0.255	28.6	22.9	0.323
statin treatment (%)	59.4	36.4	**<0.001**	38.2	52.6	**0.036**			
Comorbidities									
DM (%)	40.5	44.3	0.549	43.1	42.1	0.888	49.5	36.0	**0.036**
hypertension (%)	79.7	76.0	0.487	80.3	76.9	0.553	85.8	70.5	**0.004**
heart failure (%)	19.5	21.5	0.702	17.1	22.0	0.382	17.5	23.3	0.261
COPD (%)	14.8	14.0	0.859	9.2	16.8	0.119	15.0	14.0	0.814
asthma (%)	2.3	1.7	0.698	1.3	2.3	0.606	3.3	0.8	0.150
renal disease (%)	53.2	39.2	**0.028**	30.3	53.5	**<0.001**	47.5	45.2	0.722
stroke/TIA/CVD (%)	29.7	19.0	0.050	26.3	23.7	0.658	33.3	16.3	**0.002**
comorbidity count	3.4 ± 1.3	3.1 ± 1.2	0.067	3.1 ± 1.2	3.4 ± 1.2	0.051	3.5 ± 1.2	3.0 ± 1.2	**0.005**
Outcome									
mortality (%)	9.4	22.3	**0.005**	22.4	12.7	**0.054**	12.5	18.6	0.185
ICU (%)	13.3	19.8	0.163	27.6	11.6	**0.002**	14.2	18.6	0.345
oxygen req (%)	48.0	76.0	**<0.001**	64.5	60.7	0.572	55.0	68.2	**0.032**
Laboratory analysis									
CT value	20.8 ± 6.5	20.7 ± 6.6	0.817	20.7 ± 6.0	20.8 ± 6.8	0.768	20.2 ± 5.7	21.2 ± 7.2	0.436
creatinine (mg/dL)	1.2 (1.0–1.7)	1.2 (0.9–1.7)	0.811	1.1 (0.9–1.5)	1.3 (1.0–1.8)	**0.020**	1.2 (1.0–1.7)	1.2 (0.9–1.7)	0.363
NT-proBNP (pg/mL)	1588 (646–4304)	975 (388–4333)	0.070	1264 (494–2527)	1532 (470–5422)	0.137	1324 (426–3427)	1566 (506–4622)	0.435
IL6 (pg/mL)	27.4 (13–78)	50.8 (22–84)	**0.029**	38.2 (15–79)	39.1 (17–83)	0.773	31.6 (12–68)	46.6 (20–97)	**0.020**
CRP (mg/dL)	6.5 ± 6.8	6.7 ± 6.2	0.593	6.6 ± 6.6	6.6 ± 6.5	0.746	5.7 ± 5.8	7.5 ± 7.0	**0.024**

**Table 3:** Comparison of patient characteristics between CAD, non-CAD and, matched non-CAD patients for vaccinated patients (left) and patients receiving statin treatment (right). Quantitative results are given as means ± standard deviation or median and interquartile range, as appropriate. BMI: body mass index, DM: diabetes mellitus, CAD: coronary artery disease, COPD: chronic obstructive pulmonary disease, TIA: transient ischemic attack, CVD: cerebrovascular disease, ICU: intensive care unit, CT: cycle threshold, BAU: binding antibody units, bold print: statistically significant.

CAD vs. Non-CAD Patients	CAD, Vaccinated	Non-CAD, Vaccinated	*p*-Value	Non-CAD, Matched Vaccinated	*p*-Value	CAD, Statin Treatment	Non-CAD, Statin Treatment	*p*-Value	Non-CAD, Statins, Matched	*p*-Value
*N*	173	445		141		120	176		66	
age (years)	79.3 ± 10.2	68.3 ± 18.9	**<0.001**	78.3 ± 11.7	0.810	77.7 ± 10.2	75.4 ± 11.8	0.175	79.6 ± 9.5	0.138
male gender (%)	59.5	55.5	0.364	60.3	0.893	65.8	55.7	0.080	59.1	0.361
BMI (kg/m^2^)	27.3 ± 5.9	26.7 ± 5.8	0.150	26.3 ± 4.9	0.143	27.7 ± 5.4	28.5 ± 6.5	0.522	28.1 ± 5.0	0.474
obesity (%)	27.8	21.6	0.120	16.8	0.028	28.6	32.9	0.450	29.3	0.920
statin treatment (%)	52.6	27.2	**<0.001**	34.8	**0.002**					
Comorbidities										
DM (%)	42.1	23.8	**<0.001**	51.1	0.116	49.5	43.9	0.354	65.2	**0.043**
hypertension (%)	76.9	51.0	**<0.001**	79.4	0.587	85.8	68.2	**<0.001**	86.4	0.921
heart failure (%)	22.0	4.5	**<0.001**	6.4	**<0.001**	17.5	6.3	**0.002**	6.1	**0.029**
COPD (%)	16.8	12.4	0.152	14.2	0.531	15.0	17.0	0.639	10.6	0.401
asthma (%)	2.3	2.0	0.822	2.1	0.912	3.3	2.3	0.581	3.0	0.911
renal disease (%)	53.5	22.7	**<0.001**	43.3	0.071	47.5	25.0	**<0.001**	43.9	0.641
stroke/TIA/CVD (%)	23.7	11.5	**<0.001**	15.6	0.075	33.3	21.0	**0.018**	22.7	0.129
comorbidity count	3.4 ± 1.2	1.3 ± 1.2	**<0.001**	2.1 ± 1.1	**<0.001**	3.5 ± 1.2	1.8 ± 1.3	**<0.001**	2.4 ± 1.2	**<0.001**
Outcome										
mortality (%)	12.7	5.2	**0.001**	6.4	0.061	12.5	8.5	0.266	9.1	0.482
ICU (%)	11.6	8.3	0.211	10.6	0.796	14.2	11.4	0.474	4.5	**0.043**
oxygen req (%)	60.7	37.6	**<0.001**	43.4	**0.002**	55.0	51.7	0.577	54.5	0.952
Laboratory analysis										
CT value	20.8 ± 6.8	20.9 ± 6.6	0.736	20.8 ± 7.0	0.917	20.2 ± 5.7	20.2 ± 6.1	0.903	21.3 ± 6.3	0.368
creatinine (mg/dL)	1.3 (1.0–1.8)	1.0 (0.8–1.3)	**<0.001**	1.2 (0.9–1.6)	0.077	1.2 (1.0–1.7)	1.1 (0.9–1.4)	**<0.001**	1.2 (0.9–1.5)	0.103
NT-proBNP (pg/mL)	1532 (470–5422)	302 (96–1140)	**<0.001**	734 (211–2039)	**<0.001**	1324 (426–3427)	384 (165–1388)	**<0.001**	534 (211–1049)	**<0.001**
IL6 (pg/mL)	39.1 (17–83)	22.1 (8–58)	**<0.001**	22.4 (9–44)	**<0.001**	31.6 (12–68)	26.6 (9–62)	0.229	27.0 (9–57)	0.430
CRP (mg/dL)	6.6 ± 6.5	5.2 ± 6.4	**<0.001**	5.2 ± 6.5	**0.005**	5.7 ± 5.8	5.5 ± 6.5	0.412	5.9 ± 7.3	0.890

## Data Availability

As personal individual information is included in the dataset, the data pertaining to this investigation are not publicly available to protect study participant privacy. However, an anonymized version will be shared upon reasonable request to the corresponding author.

## Abbreviations

(a)OR, (a)HR	(adjusted) odds ratio, (adjusted) hazard ratio.
BAU/ml	Binding antibody units/ml.
BMI	Body mass index.
CAD	Coronary artery disease.
CI	Confidence interval.
COPD	Chronic obstructive pulmonary disease.
COVID-19	Coronavirus disease 2019.
CRP	C-reactive protein.
CVD	Cerebrovascular disease.
ICU	Intensive care unit.
IL6	Interleukin 6.
IQR	Interquartile range.
PCR	Polymerase chain reaction.
SARS-CoV-2	Severe acute respiratory syndrome coronavirus 2.
STEMI	ST elevation myocardial infarction.
TIA	Transient ischemic attack.
